# The Spread of Spectrin in Ataxia and Neurodegenerative Disease

**Published:** 2021

**Authors:** Jon S. Morrow, Michael C. Stankewich

**Affiliations:** 1 Department of Pathology, Yale School of Medicine, New Haven, CT 06520, USA; 2 Molecular & Cellular Developmental Biology, Yale University, New Haven, CT 06520, USA

**Keywords:** Membrane-associated periodic skeleton, Calpain, Proteolysis, SPTB, SPTBN1, SPTBN2, SPTAN1, Alzheimer’s, SCA5, Parkinson’s, Neurodegeneration, Proteostasis, RTK signaling, Calcium signaling, HIPPO/YAP signaling

## Abstract

Experimental and hereditary defects in the ubiquitous scaffolding proteins of the spectrin gene family cause an array of neuropathologies. Most recognized are ataxias caused by missense, deletions, or truncations in the SPTBN2 gene that encodes beta III spectrin. Such mutations disrupt the organization of post-synaptic receptors, their active transport through the secretory pathway, and the organization and dynamics of the actin-based neuronal skeleton. Similar mutations in SPTAN1 that encodes alpha II spectrin cause severe and usually lethal neurodevelopmental defects including one form of early infantile epileptic encephalopathy type 5 (West syndrome). Defects in these and other spectrins are implicated in degenerative and psychiatric conditions. In recent published work, we describe in mice a novel variant of alpha II spectrin that results in a progressive ataxia with widespread neurodegenerative change. The action of this variant is distinct, in that rather than disrupting a constitutive ligand-binding function of spectrin, the mutation alters its response to calcium and calmodulin-regulated signaling pathways including its response to calpain activation. As such, it represents a novel spectrinopathy that targets a key regulatory pathway where calcium and tyrosine kinase signals converge. Here we briefly discuss the various roles of spectrin in neuronal processes and calcium activated regulatory inputs that control its participation in neuronal growth, organization, and remodeling. We hypothesize that damage to the neuronal spectrin scaffold may be a common final pathway in many neurodegenerative disorders. Targeting the pathways that regulate spectrin function may thus offer novel avenues for therapeutic intervention.

## Introduction

**Figure 1:**
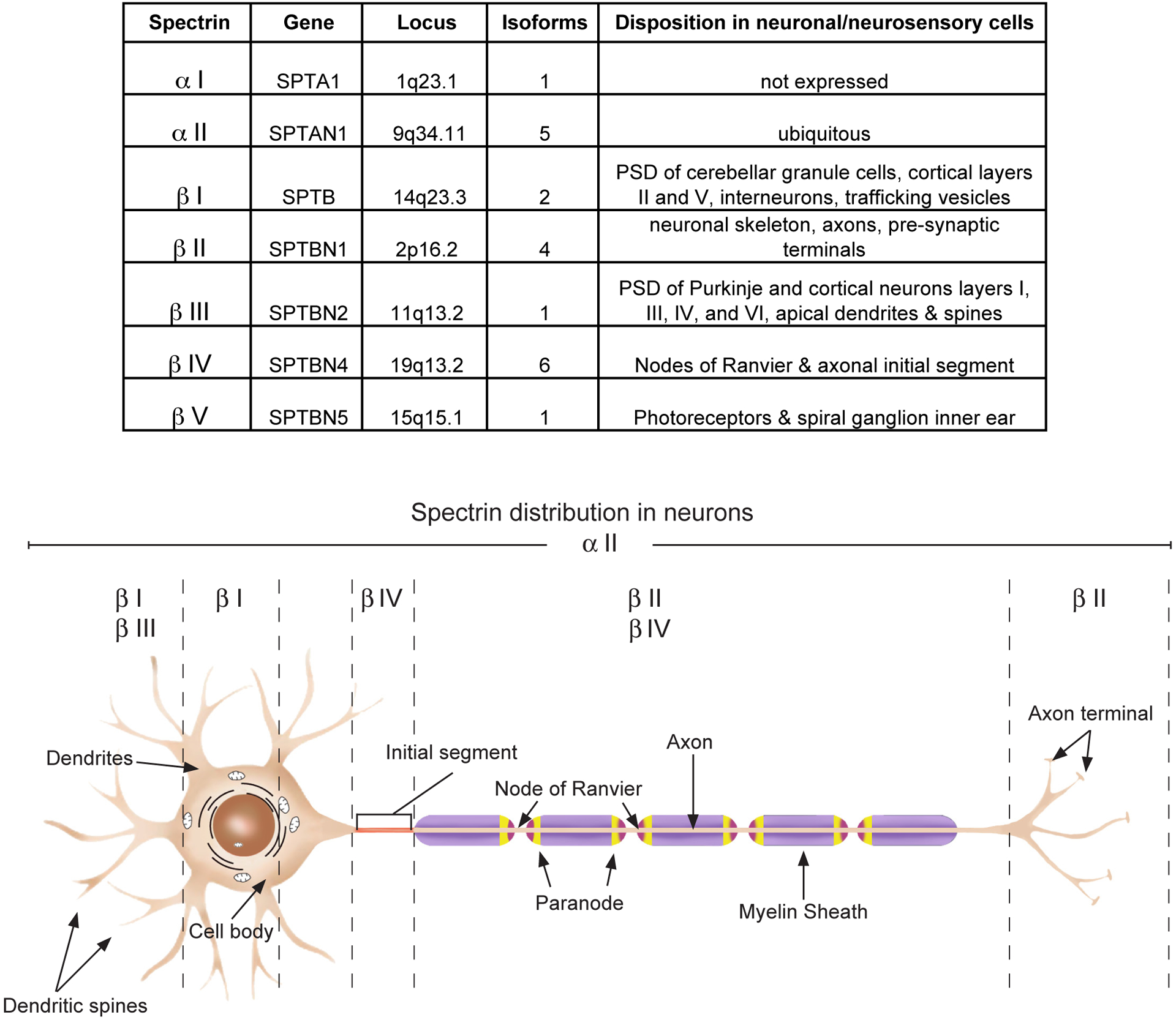
Disposition of the spectrin gene family in neuronal and neurosensory cells and tissues.

**Figure 2:**
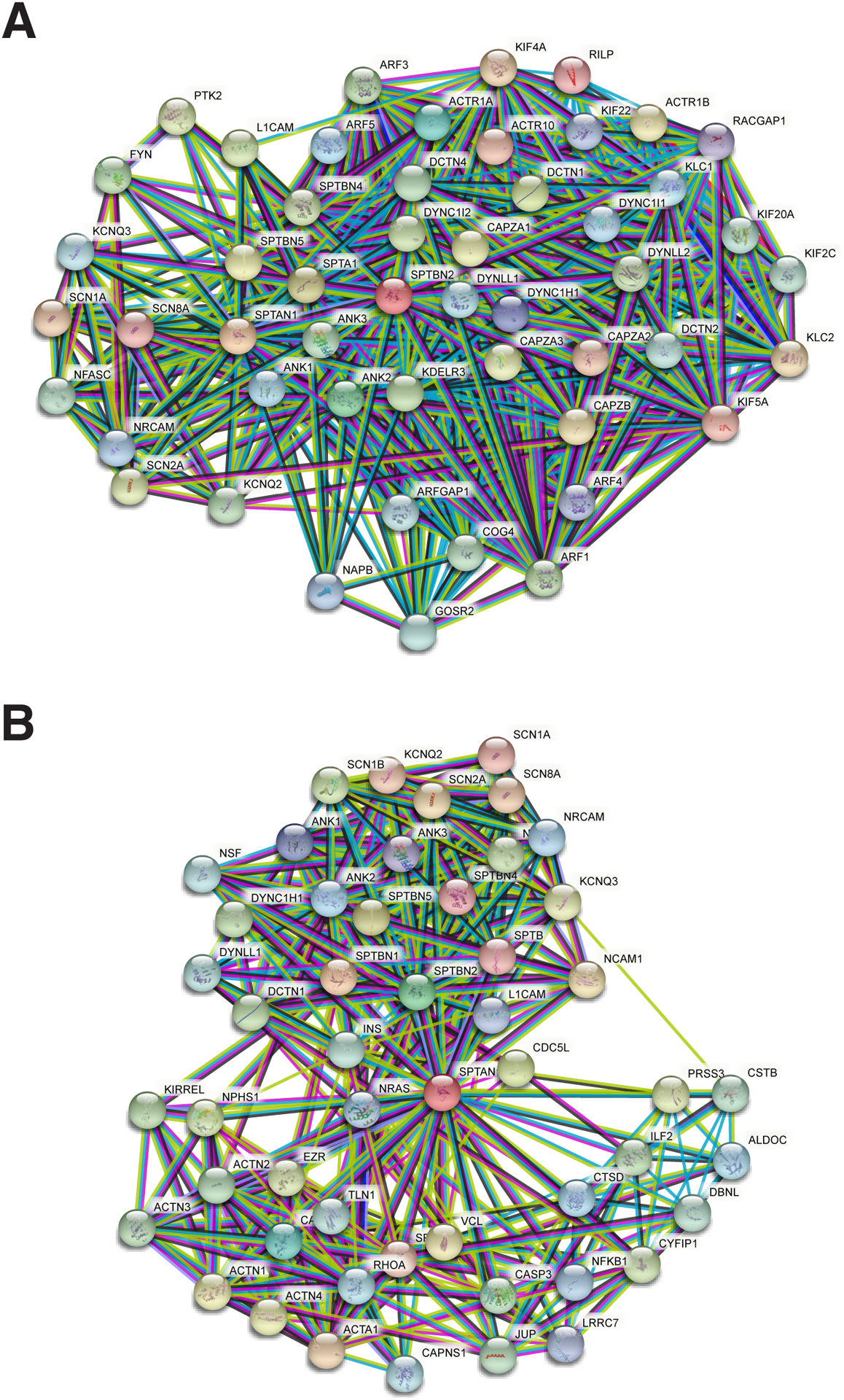
Genome-wide Interaction map of spectrin in humans. **(A)** Interaction map of βIII spectrin. **(B)** Interaction map of αII spectrin. The fifty interacting genes with the highest confidence score (>0.9) are represented in each diagram. The edges represent known protein-protein interactions (not necessarily directly bound). Nodes are the respective proteins centered on each spectrin. Edge colors are: purple, experimentally determined; light blue, curated databases; green, gene neighborhood associations. Note the significant interactions of spectrin with cytoskeletal elements, motors of intracellular transport; many ion channels and transporters; components of the Golgi apparatus and the secretory and endocytic pathways; and various receptor tyrosine kinases and adapter proteins. Generated by Strings V11 [78].

Since its recognition five decades ago as a major component of the erythrocyte’s cortical membrane skeleton, our understanding of spectrin has evolved to include recognition of its ubiquitous presence in probably all animal cells and its role in surprisingly diverse biological functions. This is perhaps most apparent in the nervous system. Seven genes encode the mammalian spectrins (Figure 1). All but one (αI spectrin) are expressed in various neuronal and neurosensory cells. Classically, spectrin is recognized as an actin filament cross-linking protein that also binds directly and through adapter proteins (e.g. ankyrin) to biologic membranes and membrane lipids. In neurons and glia, spectrin forms a caricature of the erythrocyte skeleton, termed the membrane-associated periodic skeleton (MPS) [1]. Beyond its canonical role as an actin binding and membrane-linking protein, the spectrins also serve other roles: i) linkage to motors of intracellular transport, myosin, dynactin and kinesin [2–5]; ii) linkage to the axonal transport of lipid and protein laden vesicles [3,4] iii) stabilization of the Golgi and endoplasmic reticulum [6–9]; iv) trafficking of selected proteins in the secretory and endocytic pathways [8,10–12]; v) upstream regulation of the HIPPO/YAP signaling pathway that guides many aspects of neuronal development and remodeling [13–16]; vi) a multivalent protein-protein interaction scaffold that organizes membrane-associated signaling ensembles [17]; and vii) a target of multiple post-translational modifications that regulate its various functions. The richness of spectrin’s direct and indirect interactions with many biologic pathways can be appreciated in genome wide interaction diagrams of any spectrin; two examples for human βIII spectrin and αII spectrin are shown in Figure 2.

## Spectrinopathies

Reflecting their diverse roles, spectrin deficiencies or defects lead to diverse neuropathology. Most studied have been the beta spectrins. Disorders in βI spectrin (SPTB) have been linked genetically to autism, learning difficulties, and spinal cord disease [18–20]. Genetic deletion of βII spectrin is embryonic lethal with loss of neural stem cells in the subventricular zone [21,22]; heterozygotes appear neurologically normal, but are prone to develop liver and gastrointestinal cancers putatively due to alterations in TGF-β/SMAD signaling [23]. Spectrin is also linked genetically to late-onset Parkinson’s disease and Lewy-body pathology [24,25] as well as other neurodevelopmental syndromes [26]. βIII spectrinopathies include spinocerebellar ataxia type 5 (SCA5) as found in afflicted decedents of Abraham Lincoln [27] and now recognized in several other pedigrees [28]. Other variants in SPTBN2 show cognitive impairment as well as ataxia (spectrin-associated autosomal recessive cerebellar ataxia type 1, SPARCA1) [29,30]. CpG hypomethylation of SPTBN2 links to attention deficits in children [31]. Animal models with genetic deletion of βIII spectrin recapitulate these conditions, with demonstrative disruption of the ER and Golgi architecture and selective mis-localization of postsynaptic proteins and excitatory amino acid transporters [11,32]. Defects in βIV spectrin disrupt axonal organization at the nodes of Ranvier and subsequently neurotransmission, and are associated with congenital myopathies and deafness [25,33]. Finally, spectrin βV is required to link myosin VIIA to trafficking vesicles; failure of this linkage leads to progressive hearing loss and blindness in Usher syndrome Type I [34] along with impairment of innervation of the organ of Corti [35].

Defects in αII spectrin also lead to neurologic pathology. Mice lacking αII spectrin are embryonic lethal due to cardiac and nervous system malformations [36]. Mice without αII spectrin in the peripheral nervous system suffer impaired neuronal excitability and axonal defects [37,38]. Human mutations in αII spectrin (*SPTAN1)* link to early infantile epileptic encephalopathy (EIEE) type 5 (West Syndrome), characterized by refractory seizures, intellectual disability, agenesis of the corpus callosum and hypomyelination [38–41]. Other SPTAN1 neurological disorders include juvenile onset hereditary motor neuropathy and hereditary spastic paraplegia [28,42–46].

In recent published work, we describe a novel variant of the murine Sptan1 gene (αII spectrin) with a substitution of Gln for Arg at codon 1098. In heterozygotes this substitution causes a progressive age-dependent ataxia with widespread neurodegeneration [47]. The action of this variant is distinct from other αII spectrin neuropathologic mutations, in that rather than directly disrupting a constitutive ligand-binding or protein-protein interaction (e.g. heterodimer formation, tetramer formation, or actin binding), the mutation alters spectrin’s susceptibility to calcium and calmodulin activated calpain proteolysis, with secondary consequences for its overall function. Beyond the pathways that modulate calpain activity [48], two factors control spectrin’s susceptibility at the substrate level to activated calpain: the calcium-calmodulin dependent exposure of its Y-G residues at position 1176–1177 [49,50], and whether Y1176 is phosphorylated [51,52]. The R1098Q variant spectrin thus represents a novel spectrinopathy that targets a key regulatory site where calcium and tyrosine kinase signal pathways converge to alter spectrin’s function. Beyond the novelty of the R1098Q mutation, the implications of this pathway for understanding other neurodegenerative disorders are significant.

## Impaired Spectrin Homeostasis: A Unifying Concept of Neuronal Injury

Inappropriate calcium signaling and activation of calcium activated neutral proteases (calpain) is implicated in a variety of neurologic or degenerative disorders. These include Alzheimer’s and Parkinson’s disease [48,53–55], aging [56,57], and traumatic brain injury [58,59]. The literature is replete with putative calpain targets, and a case can be made that many of these contribute to any given pathology. However, αII spectrin is a major target of calpain attack in all of these conditions, and its cleavage has been widely used as a sensitive measure of neuronal remodeling or neurodegeneration or neurotoxicity. It is an early event in the generation of dark Purkinje cells [60], and calpain-generated breakdown products of spectrin appear in association with amyloid-beta (Aβ deposits and neurofibrillary tangles in Alzheimer’s disease patients [61,62]. Spectrin is also a component of the Lewy bodies found in Parkinson’s disease patients, although its state of proteolytic cleavage in the Lewy bodies is undetermined [63]. While the association of spectrin breakdown with disease could simply reflect the end-stage consequences of neuronal injury, we believe this is unlikely based on the global involvement of spectrin in so many cellular processes, and particularly based on our findings with the αII spectrin R1098Q variant [47]. This variant informs us that up-regulation alone of spectrin’s sensitivity to calpain cleavage is sufficient to induce widespread neurodegenerative change and lethal cell injury. It is important to emphasize, this effect is mediated by an enhancement of spectrin’s intrinsic sensitivity to cleavage, not by a global activation of calpain or enhanced Ca^++^ signaling. While mutations in spectrin remain a rare cause of neurologic disease, processes that perturb intracellular calcium homeostasis and calpain activity are not rare, and accompany many neurodegenerative and other disorders as noted above. It is thus likely that enhanced cellular calpain activity alone, acting on wild-type αII spectrin, will phenocopy the consequences of the R1098Q variant. It will be important in the future to confirm this by determining that a reduction in calpain activity can rescue the R1098Q phenotype. Regardless, the totality of this data suggests that disruption of the spectrin scaffold in neuronal or neurosensory cells may be a common final pathway of neurodegeneration or malfunction.

## A Path to Therapy

To the extent that disruption of the neuronal spectrin scaffold is an important factor in the progression of neurologic disease, strategies designed to ameliorate spectrin dysfunction may offer new routes for therapeutic intervention. Inhibition of calpain activity has long been recognized as a potential therapeutic target, and pharmacological calpain inhibitors [64] or over-expression of the natural calpain inhibitor calpastatin [65] prevents or reduces neurodegeneration in murine models. However, recognition that the effects of calpain on the spectrin scaffold can also be regulated at the substrate level opens the door to novel and possibly more specific interventions. One strategy might focus on the phosphorylation of the tyrosine at codon 1176 (Y1176) by a Src family kinase, a post-translational modification that also blocks the calpain cleavage of αII spectrin [51,52]. Designing a therapeutic strategy that either activates such a kinase or inhibits a relevant phosphatase could offer benefit and greater specificity than global calpain suppression. Recently, one study found that Trodusquemine, an inhibitor of tyrosine phosphatase PTB1B that is currently in phase 1–2 clinical trials for obesity, restored synaptic plasticity and improved cognitive function in a murine model of Alzheimer’s disease [66,67]. While spectrin was not identified as a target of PTP1B in this study, the enzyme is abundant in brain and any blockage of calpain processing of spectrin by tyrosine phosphorylation at residue 1176 would also impair synaptic plasticity [68,69].

Beyond the action of calpain, the spectrin scaffold as a major organizing and structural hub offers many other putative targets for therapeutic intervention. In Parkinson’s disease models, phosphorylated α-synuclein binds preferentially to spectrin, which removes potentially toxic α-synuclein aggregates [70]. However, the binding of monomeric or oligomeric α-synuclein to spectrin disrupts spectrin-actin dynamics and the membrane-associated periodic skeleton. As seen in most pedigrees with spectrin mutations related to its actin-binding function, disruption of the MPS alone is significantly pathologic. It is interesting to speculate that the end-stage injury in Parkinson’s disease could be related as much to α-synuclein induced damage to the MPS as to the accumulation of α-synuclein aggregates. Since α-synuclein’s affinity for spectrin is enhanced in the Drosophila model by its phosphorylation at serine 129, therapies that limit this phosphorylation might stabilize the MPS and enhance neuronal survival.

There are many other regulated interactions of the spectrin scaffold with its various partners, and more no doubt await discovery. Some examples include complex allosteric interactions that link membrane binding to its self-association properties [71,72], post-translational regulation controlling its interaction with calmodulin-regulated kinases (CaMKII) [73–75], its control of calcium-regulated exocytosis [76], phosphorylation control of its stabilization of specific organelles [6,7], and its interaction with long non-coding RNA’s (lncRNA) responsible for activity-dependent synaptic plasticity in hippocampal neurons [77]. If indeed as we have postulated that damage to the spectrin scaffold is a crucial final pathway in a broad spectrum of neuronal pathologies, then strategies designed to stabilize this structure may offer truly new approaches to their treatment.

## Conclusion

From its humble beginnings as a cytoskeletal protein originally thought to be unique to the red cell, the spectrin scaffold has emerged as a central component of diverse signaling systems and a crucial member of pathways that control cellular organization, size, and function. This is most apparent in the nervous system. Defects in the spectrin scaffold lead to diverse neurodevelopmental and acquired disorders. The recent identification of an unusual αII spectrin defect in a murine model that renders it uniquely hypersensitive to calcium-mediated calpain processing indicates that damage to the spectrin skeleton alone is sufficient to generate a severe ataxic phenotype with widespread neurodegenerative change [47]. Increasing evidence indicates that diverse neurodegenerative and acquired conditions including Alzheimer’s disease, Parkinson’s disease, several ataxias, and traumatic brain injury all involve damage to the spectrin scaffold. Thus, an attractive hypothesis is that disruption of the neuronal spectrin scaffold represents a common end-stage event in such disorders. Therapeutic strategies focused on preservation of spectrin’s function may thus offer novel approaches to the treatment of many neurodegenerative conditions.

## References

[1] Xu K, Zhong G, Zhuang X. Actin, spectrin, and associated proteins form a periodic cytoskeletal structure in axons. Science. 2013 Jan 25; 339(6118):452–6. 23239625 10.1126/science.1232251 PMC3815867

[2] Deng H, Wang W, Yu J, Zheng Y, Qing Y, Pan D. Spectrin regulates Hippo signaling by modulating cortical actomyosin activity. Elife. 2015 Mar 31; 4:e06567. 10.7554/eLife.06567 PMC4412106 25826608

[3] Muresan V, Stankewich MC, Steffen W, Morrow JS, Holzbaur EL, Schnapp BJ. Dynactin-dependent, dynein-driven vesicle transport in the absence of membrane proteins: a role for spectrin and acidic phospholipids. Molecular Cell. 2001 Jan 1; 7(1):173–83. 11172722 10.1016/s1097-2765(01)00165-4

[4] Holleran EA, Ligon LA, Tokito M, Stankewich MC, Morrow JS, Holzbaur EL. βIII spectrin binds to the Arp1 subunit of dynactin. Journal of Biological Chemistry. 2001 Sep 28; 276(39):36598–605. 11461920 10.1074/jbc.M104838200

[5] Takeda S, Yamazaki H, Seog DH, Kanai Y, Terada S, Hirokawa N. Kinesin superfamily protein 3 (KIF3) motor transports fodrin-associating vesicles important for neurite building. The Journal of Cell Biology. 2000 Mar 20; 148(6):1255–66. 10725338 10.1083/jcb.148.6.1255 PMC2174314

[6] Siddhanta A, Radulescu A, Stankewich MC, Morrow JS, Shields D. Fragmentation of the golgi apparatus: a role for βIII spectrin and synthesis of phosphatidylinositol 4, 5-bisphosphate. Journal of Biological Chemistry. 2003 Jan 17; 278(3):1957–65. 12411436 10.1074/jbc.M209137200

[7] Godi A, Santone I, Pertile P, Devarajan P, Stabach PR, Morrow JS, ADP ribosylation factor regulates spectrin binding to the Golgi complex. Proceedings of the National Academy of Sciences. 1998 Jul 21; 95(15):8607–12. 10.1073/pnas.95.15.8607 PMC21123 9671725

[8] Salcedo-Sicilia L, Granell S, Jovic M, Sicart A, Mato E, Johannes L, βIII spectrin regulates the structural integrity and the secretory protein transport of the Golgi complex. Journal of Biological Chemistry. 2013 Jan 25; 288(4):2157–66. 23233669 10.1074/jbc.M112.406462 PMC3554888

[9] Stankewich MC, William TT, Peters LL, Ch’ng Y, John KM, Stabach PR, A widely expressed βIII spectrin associated with Golgi and cytoplasmic vesicles. Proceedings of the National Academy of Sciences. 1998 Nov 24;95(24):14158–63. 10.1073/pnas.95.24.14158 PMC24343 9826670

[10] Devarajan P, Stabach PR, De Matteis MA, Morrow JS. Na, K-ATPase transport from endoplasmic reticulum to Golgi requires the Golgi spectrin–ankyrin G119 skeleton in Madin Darby canine kidney cells. Proceedings of the National Academy of Sciences. 1997 Sep 30; 94(20):10711–6. 10.1073/pnas.94.20.10711 PMC23456 9380700

[11] Stankewich MC, Gwynn B, Ardito T, Ji L, Kim J, Robledo RF, Targeted deletion of βIII spectrin impairs synaptogenesis and generates ataxic and seizure phenotypes. Proceedings of the National Academy of Sciences. 2010 Mar 30; 107(13):6022–7. 10.1073/pnas.1001522107 PMC2851889 20231455

[12] De Matteis MA, Morrow JS. Spectrin tethers and mesh in the biosynthetic pathway. Journal of Cell Science. 2000 Jul 1; 113(13):2331–43. 10852813 10.1242/jcs.113.13.2331

[13] Deng H, Yang L, Wen P, Lei H, Blount P, Pan D. Spectrin couples cell shape, cortical tension, and Hippo signaling in retinal epithelial morphogenesis. Journal of Cell Biology. 2020 Apr 6; 219(4). 10.1083/jcb.201907018 PMC7147103 32328630

[14] Fletcher GC, Elbediwy A, Khanal I, Ribeiro PS, Tapon N, Thompson BJ. The Spectrin cytoskeleton regulates the Hippo signalling pathway. The EMBO Journal. 2015 Apr 1; 34(7):940–54. 25712476 10.15252/embj.201489642 PMC4388601

[15] Antón IM, Wandosell F. WIP, YAP/TAZ and Actin Connections Orchestrate Development and Transformation in the Central Nervous System. Frontiers in Cell and Developmental Biology. 2021 Jun 14; 9:1439. 10.3389/fcell.2021.673986 PMC8237755 34195190

[16] Sahu MR, Mondal AC. The emerging role of Hippo signaling in neurodegeneration. Journal of Neuroscience Research. 2020 May; 98(5):796–814. 31705587 10.1002/jnr.24551

[17] Machnicka B, Czogalla A, Hryniewicz-Jankowska A, Bogusławska DM, Grochowalska R, Heger E, Spectrins: a structural platform for stabilization and activation of membrane channels, receptors and transporters. Biochimica et Biophysica Acta (BBA)-Biomembranes. 2014 Feb 1; 1838(2):620–34. 23673272 10.1016/j.bbamem.2013.05.002

[18] Griswold AJ, Ma D, Sacharow SJ, Robinson JL, Jaworski JM, Wright HH, A de novo 1.5 Mb microdeletion on chromosome 14q23. 2–23.3 in a patient with autism and spherocytosis. Autism Research. 2011 Jun; 4(3):221–7. 21360829 10.1002/aur.186 PMC3110642

[19] Lybaek H, Øyen N, Fauske L, Houge G. A 2.1 Mb deletion adjacent but distal to a 14q21q23 paracentric inversion in a family with spherocytosis and severe learning difficulties. Clinical Genetics. 2008 Dec; 74(6):553–9. 18717686 10.1111/j.1399-0004.2008.01072.x

[20] McCann SR, Jacob HS. Spinal cord disease in hereditary spherocytosis: report of two cases with a hypothesized common mechanism for neurologic and red cell abnormalities. Blood. 1976 Aug 1; 48(2):259–63. 949548

[21] Tang Y, Katuri V, Dillner A, Mishra B, Deng CX, Mishra L. Disruption of transforming growth factor-β signaling in ELF β-spectrin-deficient mice. Science. 2003 Jan 24; 299(5606):574–7. 12543979 10.1126/science.1075994

[22] Golestaneh N, Tang Y, Katuri V, Jogunoori W, Mishra L, Mishra B. Cell cycle deregulation and loss of stem cell phenotype in the subventricular zone of TGF-β adaptor elf−/− mouse brain. Brain Research. 2006 Sep 7; 1108(1):45–53. 16884701 10.1016/j.brainres.2006.05.113

[23] Kim SS, Shetty K, Katuri V, Kitisin K, Baek HJ, Tang Y, TGF-β signaling pathway inactivation and cell cycle deregulation in the development of gastric cancer: Role of the β-spectrin, ELF. Biochemical and Biophysical Research Communications. 2006 Jun 16; 344(4):1216–23. 16650383 10.1016/j.bbrc.2006.03.236 PMC4211257

[24] Peuralinna T, Myllykangas L, Oinas M, Nalls MA, Keage HA, Isoviita VM, Genome-wide association study of neocortical Lewy-related pathology. Annals of Clinical and Translational Neurology. 2015 Sep; 2(9):920–31. 26401513 10.1002/acn3.231 PMC4574809

[25] Knierim E, Gill E, Seifert F, Morales-Gonzalez S, Unudurthi SD, Hund TJ, A recessive mutation in beta-IV-spectrin (SPTBN4) associates with congenital myopathy, neuropathy, and central deafness. Human Genetics. 2017 Jul; 136(7):903–10. 28540413 10.1007/s00439-017-1814-7

[26] Cousin MA, Creighton BA, Breau KA, Spillmann RC, Torti E, Dontu S, Pathogenic SPTBN1 variants cause an autosomal dominant neurodevelopmental syndrome. Nature Genetics. 2021 Jul 1:1–6. 10.1038/s41588-021-00886-z PMC8273149 34211179

[27] Ikeda Y, Dick KA, Weatherspoon MR, Gincel D, Armbrust KR, Dalton JC, Spectrin mutations cause spinocerebellar ataxia type 5. Nature Genetics. 2006 Feb; 38(2):184–90. 16429157 10.1038/ng1728

[28] Syrbe S, Harms FL, Parrini E, Montomoli M, Mütze U, Helbig KL, Delineating SPTAN1 associated phenotypes: from isolated epilepsy to encephalopathy with progressive brain atrophy. Brain. 2017 Sep 1; 140(9):2322–36. 29050398 10.1093/brain/awx195 PMC6248409

[29] Yıldız Bölükbaşı E, Afzal M, Mumtaz S, Ahmad N, Malik S, Tolun A. Progressive SCAR14 with unclear speech, developmental delay, tremor, and behavioral problems caused by a homozygous deletion of the SPTBN2 pleckstrin homology domain. American Journal of Medical Genetics Part A. 2017 Sep; 173(9):2494–9. 28636205 10.1002/ajmg.a.38332

[30] Lise S, Clarkson Y, Perkins E, Kwasniewska A, Sadighi Akha E, Parolin Schnekenberg R, Recessive mutations in SPTBN2 implicate β-III spectrin in both cognitive and motor development. PLoS Genetics. 2012 Dec 6; 8(12):e1003074. 23236289 10.1371/journal.pgen.1003074 PMC3516553

[31] Li SC, Kuo HC, Huang LH, Chou WJ, Lee SY, Chan WC, DNA Methylation in LIME1 and SPTBN2 Genes Is Associated with Attention Deficit in Children. Children. 2021 Feb; 8(2):92. 33572947 10.3390/children8020092 PMC7912017

[32] Perkins EM, Clarkson YL, Sabatier N, Longhurst DM, Millward CP, Jack J, Loss of β-III spectrin leads to Purkinje cell dysfunction recapitulating the behavior and neuropathology of spinocerebellar ataxia type 5 in humans. Journal of Neuroscience. 2010 Apr 7; 30(14):4857–67. 20371805 10.1523/JNEUROSCI.6065-09.2010 PMC2857506

[33] Buelow M, Süßmuth D, Smith LD, Aryani O, Castiglioni C, Stenzel W, Novel bi-allelic variants expand the SPTBN4-related genetic and phenotypic spectrum. European Journal of Human Genetics. 2021 Mar 26:1–8. 10.1038/s41431-021-00846-5 PMC8298470 33772159

[34] Papal S, Cortese M, Legendre K, Sorusch N, Dragavon J, Sahly I, The giant spectrin βV couples the molecular motors to phototransduction and Usher syndrome type I proteins along their trafficking route. Human Molecular Genetics. 2013 Sep 15; 22(18):3773–88. 23704327 10.1093/hmg/ddt228

[35] Stankewich MC, Bai J-P, Stabach PR, Khan S, Tan WJT, Surguchev A, Outer hair cell function is normal in beta V spectrin knockout mice. BioRxiv. 2021 Aug 5. 10.1016/j.heares.2022.108564 35864018

[36] Stankewich MC, Cianci CD, Stabach PR, Ji L, Nath A, Morrow JS. Cell organization, growth, and neural and cardiac development require αII-spectrin. Journal of Cell Science. 2011 Dec 1; 124(23):3956–66. 22159418 10.1242/jcs.080374 PMC3244980

[37] Huang CY, Zhang C, Zollinger DR, Leterrier C, Rasband MN. An αII spectrin-based cytoskeleton protects large-diameter myelinated axons from degeneration. Journal of Neuroscience. 2017 Nov 22; 37(47):11323–34. 29038243 10.1523/JNEUROSCI.2113-17.2017 PMC5700418

[38] Wang Y, Ji T, Nelson AD, Glanowska K, Murphy GG, Jenkins PM, Critical roles of αII spectrin in brain development and epileptic encephalopathy. The Journal of Clinical Investigation. 2018 Feb 1; 128(2):760–73. 29337302 10.1172/JCI95743 PMC5785268

[39] Saitsu H, Tohyama J, Kumada T, Egawa K, Hamada K, Okada I, Dominant-negative mutations in α-II spectrin cause West syndrome with severe cerebral hypomyelination, spastic quadriplegia, and developmental delay. The American Journal of Human Genetics. 2010 Jun 11; 86(6):881–91. 20493457 10.1016/j.ajhg.2010.04.013 PMC3032058

[40] Writzl K, Primec ZR, Stražišar BG, Osredkar D, Pečarič-Meglič N, Kranjc BS, Early onset West syndrome with severe hypomyelination and coloboma-like optic discs in a girl with SPTAN1 mutation. Epilepsia. 2012 Jun; 53(6):e106–10. 22429196 10.1111/j.1528-1167.2012.03437.x

[41] Rapaccini V, Esposito S, Strinati F, Allegretti M, Manfroi E, Miconi F, A Child with a c. 6923_6928dup (p. Arg2308_Met2309dup) SPTAN1 mutation associated with a severe early infantile epileptic encephalopathy. International journal of Molecular Sciences. 2018 Jul; 19(7):1976. 10.3390/ijms19071976 PMC6073498 29986434

[42] Tohyama J, Nakashima M, Nabatame S, Miyata R, Rener-Primec Z, Kato M, SPTAN1 encephalopathy: distinct phenotypes and genotypes. Journal of Human Genetics. 2015 Apr; 60(4):167–73. 25631096 10.1038/jhg.2015.5

[43] Hamdan FF, Saitsu H, Nishiyama K, Gauthier J, Dobrzeniecka S, Spiegelman D, Identification of a novel in-frame de novo mutation in SPTAN1 in intellectual disability and pontocerebellar atrophy. European Journal of Human Genetics. 2012 Jul; 20(7):796–800. 22258530 10.1038/ejhg.2011.271 PMC3376261

[44] Beijer D, Deconinck T, De Bleecker JL, Dotti MT, Malandrini A, Urtizberea JA, Nonsense mutations in alpha-II spectrin in three families with juvenile onset hereditary motor neuropathy. Brain. 2019 Sep 1;142(9):2605–16. 31332438 10.1093/brain/awz216

[45] Gartner V, Markello TC, Macnamara E, De Biase A, Thurm A, Joseph L, Novel variants in SPTAN1 without epilepsy: an expansion of the phenotype. American Journal of Medical Genetics Part A. 2018 Dec; 176(12):2768–76. 30548380 10.1002/ajmg.a.40628 PMC11157598

[46] Leveille E, Estiar MA, Krohn L, Spiegelman D, Dionne-Laporte A, Dupré N, SPTAN1 variants as a potential cause for autosomal recessive hereditary spastic paraplegia. Journal of Human Genetics. 2019 Nov; 64(11):1145–51. 31515523 10.1038/s10038-019-0669-2

[47] Miazek A, Zalas M, Skrzymowska J, Bogin BA, Grzymajło K, Goszczynski TM, Age-dependent ataxia and neurodegeneration caused by an αII spectrin mutation with impaired regulation of its calpain sensitivity. Scientific Reports. 2021 Mar 31; 11(1):1–8. 33790315 10.1038/s41598-021-86470-1 PMC8012654

[48] Mahaman YA, Huang F, Kessete Afewerky H, Maibouge TM, Ghose B, Wang X. Involvement of calpain in the neuropathogenesis of Alzheimer’s disease. Medicinal Research Reviews.2019 Mar; 39(2):608–30. 30260518 10.1002/med.21534 PMC6585958

[49] Harris AS, Croall DE, Morrow JS. Calmodulin regulates fodrin susceptibility to cleavage by calciumdependent protease I. Journal of Biological Chemistry. 1989 Oct 15; 264(29):17401–8. 2551900

[50] Harris AS, Morrow JS. Calmodulin and calciumdependent protease I coordinately regulate the interaction of fodrin with actin. Proceedings of the National Academy of Sciences. 1990 Apr 1; 87(8):3009–13. 10.1073/pnas.87.8.3009 PMC53823 2326262

[51] Nedrelow JH, Cianci CD, Morrow JS. C-Src binds αII Spectrin’s Src homology 3 (SH3) domain and blocks calpain susceptibility by phosphorylating Tyr1176. Journal of Biological Chemistry. 2003 Feb 28; 278(9):7735–41. 12446661 10.1074/jbc.M210988200

[52] Nicolas G, Fournier CM, Galand C, Malbert-Colas L, Bournier O, Kroviarski Y, Tyrosine phosphorylation regulates alpha II spectrin cleavage by calpain. Molecular and Cellular Biology. 2002 May 15; 22(10):3527–36. 11971983 10.1128/MCB.22.10.3527-3536.2002 PMC133798

[53] Ahmad F, Das D, Kommaddi RP, Diwakar L, Gowaikar R, Rupanagudi KV, Isoform-specific hyperactivation of calpain-2 occurs presymptomatically at the synapse in Alzheimer’s disease mice and correlates with memory deficits in human subjects. Scientific Reports. 2018 Sep 3; 8(1):1–6. 30177812 10.1038/s41598-018-31073-6 PMC6120938

[54] Muruzheva ZM, Traktirov DS, Zubov AS, Pestereva NS, Tikhomirova MS, Karpenko MN. Calpain activity in plasma of patients with essential tremor and Parkinson’s disease: a pilot study. Neurological Research. 2021 Apr 3; 43(4):314–20. 33729106 10.1080/01616412.2020.1854004

[55] Yamashima T Reconsider Alzheimer’s disease by the ‘calpain–cathepsin hypothesis’—A perspective review. Progress in Neurobiology. 2013 Jun 1; 105:1–23. 23499711 10.1016/j.pneurobio.2013.02.004

[56] Zaidi A Plasma membrane Ca2+-ATPases: Targets of oxidative stress in brain aging and neurodegeneration. World Journal of Biological Chemistry. 2010 Sep 26; 1(9):271. 21537484 10.4331/wjbc.v1.i9.271 PMC3083975

[57] Hajieva P, Kuhlmann C, Luhmann HJ, Behl C. Impaired calcium homeostasis in aged hippocampal neurons. Neuroscience Letters. 2009 Feb 20; 451(2):119–23. 19073233 10.1016/j.neulet.2008.11.068

[58] Gan ZS, Stein SC, Swanson R, Guan S, Garcia L, Mehta D, Blood biomarkers for traumatic brain injury: a quantitative assessment of diagnostic and prognostic accuracy. Frontiers in Neurology. 2019 Apr 26; 10:446. 31105646 10.3389/fneur.2019.00446 PMC6498532

[59] Wang Y, Liu Y, Lopez D, Lee M, Dayal S, Hurtado A, Protection against TBI-induced neuronal death with post-treatment with a selective calpain-2 inhibitor in mice. Journal of Neurotrauma. 2018 Jan 1; 35(1):105–17. 28594313 10.1089/neu.2017.5024 PMC5757088

[60] Mansouri B, Henne WM, Oomman SK, Bliss R, Attridge J, Finckbone V, Involvement of calpain in AMPA-induced toxicity to rat cerebellar Purkinje neurons. European Journal of Pharmacology. 2007 Feb 28; 557(2–3):106–14. 17188264 10.1016/j.ejphar.2006.11.032

[61] Sihag RK, Cataldo AM. Brain β-spectrin is a component of senile plaques in Alzheimer’s disease. Brain research. 1996 Dec 16; 743(1–2):249–57. 9017252 10.1016/s0006-8993(96)01058-x

[62] Czogalla A, Sikorski AF. Spectrin and calpain: a ‘target’and a ‘sniper’in the pathology of neuronal cells. Cellular and Molecular Life Sciences CMLS. 2005 Sep; 62(17):1913–24. 15990959 10.1007/s00018-005-5097-0 PMC11139101

[63] Leverenz JB, Umar I, Wang Q, Montine TJ, McMillan PJ, Tsuang DW, Proteomic identification of novel proteins in cortical lewy bodies. Brain Pathology. 2007 Apr;17(2):139–45. 17388944 10.1111/j.1750-3639.2007.00048.x PMC8095629

[64] Hassen GW, Kesner L, Stracher A, Shulman A, Rockenstein E, Mante M, Effects of novel calpain inhibitors in transgenic animal model of Parkinson’s disease/dementia with Lewy bodies. Scientific Reports. 2018 Dec 27; 8(1):1–0. 30591714 10.1038/s41598-018-35729-1 PMC6308237

[65] Rao MV, McBrayer MK, Campbell J, Kumar A, Hashim A, Sershen H, Specific calpain inhibition by calpastatin prevents tauopathy and neurodegeneration and restores normal lifespan in tau P301L mice. Journal of Neuroscience. 2014 Jul 9; 34(28):9222–34. 25009256 10.1523/JNEUROSCI.1132-14.2014 PMC4087203

[66] Ricke KM, Cruz SA, Qin Z, Farrokhi K, Sharmin F, Zhang L, Neuronal protein tyrosine phosphatase 1B hastens amyloid β-associated Alzheimer’s disease in mice. Journal of Neuroscience. 2020 Feb 12; 40(7):1581–93. 31915254 10.1523/JNEUROSCI.2120-19.2019 PMC7044730

[67] Zhang L, Qin Z, Sharmin F, Lin W, Ricke KM, Zasloff M, Tyrosine phosphatase PTP1B impairs presynaptic NMDA receptor-mediated plasticity in a mouse model of Alzheimer’s disease. Neurobiology of Disease. 2021 May 24:105402. 10.1016/j.nbd.2021.105402 34044147

[68] Lynch G, Baudry M. Brain spectrin, calpain and long-term changes in synaptic efficacy. Brain Research Bulletin. 1987 Jun 1; 18(6):809–15. 3040193 10.1016/0361-9230(87)90220-6

[69] Glantz SB, Cianci CD, Iyer R, Pradhan D, Wang KK, Morrow JS. Sequential degradation of αII and βII spectrin by calpain in glutamate or maitotoxin-stimulated cells. Biochemistry. 2007 Jan 16; 46(2):502–13. 17209560 10.1021/bi061504y PMC2825692

[70] Ordonez DG, Lee MK, Feany MB. α-synuclein induces mitochondrial dysfunction through spectrin and the actin cytoskeleton. Neuron. 2018 Jan 3; 97(1):108–24. 29249285 10.1016/j.neuron.2017.11.036 PMC5755717

[71] Giorgi M, Cianci CD, Gallagher PG, Morrow JS. Spectrin oligomerization is cooperatively coupled to membrane assembly: a linkage targeted by many hereditary hemolytic anemias?. Experimental and Molecular Pathology. 2001 Jun 1; 70(3):215–30. 11418000 10.1006/exmp.2001.2377

[72] Cianci CD, Giorgi M, Morrow JS. Phosphorylation of ankyrin down-regulates its cooperative interaction with spectrin and protein 3. Journal of Cellular Biochemistry. 1988 Jul; 37(3):301–15. 2970468 10.1002/jcb.240370305

[73] Kline CF, Wright PJ, Koval OM, Zmuda EJ, Johnson BL, Anderson ME, βIV-Spectrin and CaMKII facilitate Kir6. 2 regulation in pancreatic beta cells. Proceedings of the National Academy of Sciences. 2013 Oct 22;110(43):17576–81. 10.1073/pnas.1314195110 PMC3808601 24101510

[74] Hund TJ, Koval OM, Li J, Wright PJ, Qian L, Snyder JS, A β IV-spectrin/CaMKII signaling complex is essential for membrane excitability in mice. The Journal of Clinical Investigation. 2010 Oct 1; 120(10):3508–19. 20877009 10.1172/JCI43621 PMC2947241

[75] Nassal DM, Patel NJ, Unudurthi SD, Shaheen R, Yu J, Mohler PJ, Ca2+/calmodulin kinase II–dependent regulation of βIV-spectrin modulates cardiac fibroblast gene expression, proliferation, and contractility. Journal of Biological Chemistry. 2021 Jun 18:100893. 10.1016/j.jbc.2021.100893 PMC8294584 34153319

[76] Houy S, Nicolas G, Momboisse F, Malacombe M, Bader MF, Vitale N, αII-spectrin controls calcium-regulated exocytosis in neuroendocrine chromaffin cells through neuronal Wiskott–Aldrich Syndrome protein interaction. IUBMB life. 2020 Apr;72(4):544–52. 31859439 10.1002/iub.2217

[77] Grinman E, Nakahata Y, Avchalumov Y, Espadas I, Swarnkar S, Yasuda R, Activity-regulated synaptic targeting of lncRNA ADEPTR mediates structural plasticity by localizing Sptn1 and AnkB in dendrites. Science Advances. 2021 Apr 1;7(16):eabf0605. 10.1126/sciadv.abf0605 PMC8051873 33863727

[78] Szklarczyk D, Gable AL, Lyon D, Junge A, Wyder S, Huerta-Cepas J, STRING v11: protein–protein association networks with increased coverage, supporting functional discovery in genome-wide experimental datasets. Nucleic Acids Research. 2019 Jan 8; 47(D1):D607–13. 30476243 10.1093/nar/gky1131 PMC6323986

